# A Quality Improvement Bundle to Reduce Central Line-Associated Bloodstream Infections in Neonatal Intensive Care Unit: An Observational Study

**DOI:** 10.3390/antibiotics14121208

**Published:** 2025-12-01

**Authors:** Chiara Poggi, Giulia Fontanelli, Martina Ciarcià, Giovanni Sassudelli, Camilla Fazi, Leonardo Fioravanti, Silvia Grassellini, Monica Piazza, Carlo Dani

**Affiliations:** 1 Division of Neonatology, Department of Mother and Child Care, Careggi University Hospital, 50141 Florence, Italy; fontanellig@aou-careggi.toscana.it (G.F.); grassellinis@aou-careggi.toscana.it (S.G.); piazzam@aou-careggi.toscana.it (M.P.); carlo.dani@unifi.it (C.D.); 2 Neonatal Intensive Care Unit, Azienda Ospedaliera di Verona, 37122 Verona, Italy; martina.ciarcia@aovr.veneto.it; 3 Ospedale San Giovanni di Dio, Azienda Sanitaria Firenze, 50137 Florence, Italy; giovanni.sassudelli@uslcentro.toscana.it; 4 Department of Neurosciences, Psychology, Drug Research and Child Health, University of Florence, 50139 Florence, Italy; camillafazi@hotmail.com

**Keywords:** CLABSI, newborn, prevention, bundle

## Abstract

**Background:** Dedicated bundles were proven to reduce CLABSIs in a neonatal intensive care unit (NICU). **Methods:** We performed an observational pre–post study to evaluate the impact of a bundle for CLABSI prevention in our NICU. All umbilical vein catheters (UVCs) and epicutaneo-caval catheters (ECCs) with dwell time > 2 days were included. The primary outcome was CLABSI rate/1000 central line days. **Results:** A total of 145 catheters (67 UVCs and 78 ECCs) and 142 catheters (65 UVCs and 77 ECCs) were inserted before and after bundle implementation, respectively. The duration of the UVC was significantly shorter before than after the bundle [4 (3–6) vs. 8 (6–11) days; *p* < 0.0001], while the duration of the ECC did not differ [10 (6–17) vs. 11 (6–19) days; *p* = 0.711]. CLABSI were less frequent after than before bundle (3.6 vs. 10.7/1000 CL days; *p* = 0.042); both UVC-related and ECC-related CLABSI were significantly reduced (0 vs. 7.2/1000 CL days, *p* = 0.015; and 4.4 vs. 12.3/1000 CL days, *p* = 0.044, respectively). The Kaplan–Meier curve for ECC-related CLABSIs showed no differences between the two periods (*p* = 0.255), but higher survival without CLABSIs after vs. before bundle was found if considering only ECC with dwell time < 14 days (*p* = 0.040). Gestational age (*p* = 0.004) and bundle (*p* = 0.026) were predictive factors for CLABSIs. Non-infective complications were significantly less frequent after than before bundle (11 vs. 20%, *p* = 0.033). **Conclusions:** Our bundle reduced the overall CLABSI rate, and both UVC- and ECC-related CLABSI occurrence. The efficacy for the reduction in ECC-related CLABSIs seems limited to the first 14 days of dwell time.

## 1. Introduction

Central line-associated bloodstream infections (CLABSIs) are the most common healthcare-associated infections in Neonatal Intensive Care Units (NICU) [1,2,3]. CLABSI in newborns are burdened with 10 to 20% mortality [4,5,6] and are associated with acute morbidity [4,5,6,7], increased length of hospitalization and healthcare costs [7,8], and adverse neurodevelopmental outcomes [9,10,11].

The implementation of dedicated bundles for the insertion and maintenance of central venous lines was found effective in reducing the incidence of both CLABSI and non-infective complications of central venous catheters (CVC) in NICUs [6,12,13,14,15]. Reinforcement of hand hygiene and implementation of skin antisepsis with chlorhexidine significantly reduced CLABSI from 26 to 11% in a large cohort of very-low-birth-weight infants [12], and a bundle including establishment of a dedicated kit or cart and semipermeable transparent dressing reduced CLABSI by 40% [12]. Other reported effective measures include multidisciplinary discussion [13], establishment of a dedicated team for the insertion of central venous lines [6,13], and use of tissue adhesives for central line stabilization [6,15].

In our third-level NICU, dedicated checklists for the insertion and maintenance of central venous lines have been available since 2017. In 2022 we decided to update the practices for the insertion and maintenance of CVC, with the aim to improve CLABSI prevention. CLABSI rate in our NICU before intervention was 10.7/1000 catheter days, which was higher than the incidence reported for centers with a similar case mix [6]. We decided to form a dedicated study group to develop a specific bundle for CLABSI prevention (Supplementary Table S1). We hypothesized that the bundle would reduce the rate of CLABSI, and we planned an observational study to evaluate this hypothesis.

## 2. Results

We enrolled 76 infants in the period before bundle and 84 infants in the period after bundle implementation. The mean gestational age did not differ between the two periods, but the proportion of newborns with gestational ages < 28 weeks was significantly higher in the period before vs. after the bundle (48 vs. 27%, *p* = 0.005). The CRIB II score on admission to the NICU and the neonatal SOFA score at the onset of CLABSI did not differ between the two study periods (Table 1). Bronchopulmonary dysplasia was significantly more frequent in the period before vs. after the bundle (29 vs. 20%, *p* = 0.037), while no differences were found in the occurrence of necrotizing enterocolitis, retinopathy of prematurity, and intraventricular hemorrhage ≥ grade 3 (Table 1).

**Figure 1:**
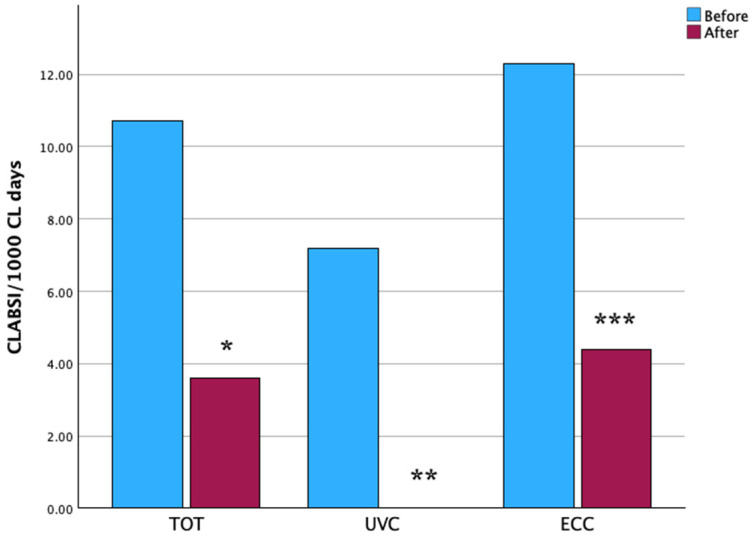
The incidence of total, UVC-related, and ECC-related CLABSI/1000 CL days in the period before and after the bundle. * *p* = 0.042, ** *p* = 0.015, *** *p* = 0.044.

In the period before the bundle, 145 CVCs, were inserted (67 UVC, and 78 ECC) while in the period after the bundle, 142 CVCs (65 UVC, and 77 ECC) were inserted. The duration of UVC was significantly shorter after than before bundle [4 (3–6) days vs. 8 (6–11); *p* < 0.0001], while the duration of ECC did not differ between the two periods [10 (6–17) vs. 11 (6–19); *p* = 0.711] (Table 2). The occurrence of CLABSI/1000 CL days was significantly lower after vs. before the bundle (10.7 vs. 3.6; *p* = 0.042); both UVC- and ECC-related CLABSI/1000 CL days were significantly less frequent after vs. before bundle (0 vs. 7.2, *p* = 0.015; and 4.4 vs. 12.3, *p* = 0.044, respectively) (Table 2, Figure 1). ECC-related CLABSI occurred after a borderline longer dwell time after vs. before the bundle (9 vs. 14 days; *p* = 0.050) (Table 2). CVC were inserted by neonatologists or fellows in neonatology, and their proportions among the operators performing the insertions did not differ between the two periods (Supplementary Table S2). The dedicated team performed insertions in the period after the bundle in 29% of cases (*p* < 0.0001 vs. before the bundle) (Table 2, Supplementary Table S2). The use of ultrasound for tip navigation and location did not significantly differ between the two periods (25 vs. 34%, *p* = 0.095).

Pathogens responsible for CLABSI were Coagulase-negative *Staphylococcus* spp. (69%), *E. coli* (12.5%), *K. pneumoniae* (12.5%), and *E. fecalis* (6%) in the period before the bundle, and Coagulase-negative *Staphylococcus* spp. (80%), *S. aureus* (10%), and *K. pneumoniae* (10%) in the period after the bundle.

**Figure 2:**
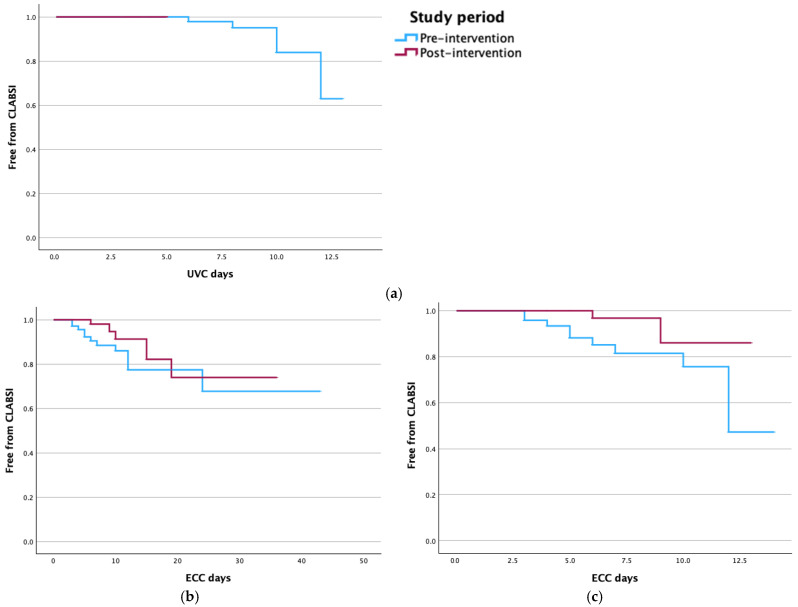
Kaplan–Meier curves for (**a**) UVC, (**b**) ECC, and (**c**) ECC with dwell time < 14 days before and after bundle.

Kaplan–Meier curves were calculated in the pre- and post-intervention periods for UVC-related CLABSI (*p* = n.a.), ECC-related CLABSI (*p* = 0.255), and ECC-related CLABSI for ECC with dwell time > 14 days (*p* = 0.040) (Figure 2).

Logistic regression analysis showed that gestational age (*p* = 0.004) and bundle implementation (*p* = 0.026) were independent predictive factors for CLABSI (Table 3).

Non-infective complications were significantly less frequent after than before bundle, on the whole (11 vs. 20%, *p* = 0.033) and for ECC (3 vs. 13%, *p* = 0.005), but not for UVC (Table 3). Non-elective removal was significantly less frequent after vs. before bundle implementation on the whole (11 vs. 22%; *p* = 0.100) and for ECC (12 vs. 26%, *p* = 0.015), but not for UVC (Table 2).

## 3. Discussion

CLABSI prevention in NICUs is crucial for improving neonatal outcomes, as the vast majority of admitted patients require one or more CVC, especially if they are born extremely preterm. Our study demonstrated that a dedicated bundle including early removal of UVC and measures targeting intraluminal and extraluminal contamination of ECC is effective in reducing the incidence of CLABSI. The total CLABSI rate fell by 66% (3.6 vs. 10.7 per 1000 CL days) after bundle implementation. The lower proportion of newborns with gestational age under 28 weeks in the post-intervention period (27% vs. 48%) may have contributed to this decrease. However, after adjustment for possible confounders, the bundle still significantly predicted CLABSI risk along with gestational age, confirming the effectiveness of our NICU’s preventive measures.

Before the bundle, UVC-related CLABSI rate was 7.2/1000 CL days, while no cases occurred in the period after bundle, likely because of the reduced dwell time of UVC (4 vs. 8 days). Our data are consistent with the previous demonstration of a positive correlation between UVC dwell time and the occurrence of CLABSI [16,17,18], with a stepwise increase from 9/1000 UVC days at 2–3 days to 42/1000 UVC days at 10 days [17]. In contrast with the previous report of 14 cases of CLABSI/1000 UVC days for a dwell time of 4–5 days [17], we reported no cases of CLABSI for the same dwell time, likely because our study showed a lower overall incidence of UVC-related CLABSI cases. Our data support the current Infusion Nurses Society guidelines and the GaveCelt/GavePed recommendations encouraging UVC removal within 4–5 days of life as a strategy for CLABSI prevention [19,20]. In contrast, a recent study reported a significant increase in CLABSI rate after the 12th day of dwell time [16], but no significant differences if UVC was maintained for 3–6 vs. 7–12 days. This might be explained by the reported lower incidence of UVC-related CLABSI of 3/1000 UVC days [16], in comparison to our period before bundle. However, while an incidence of 3.7/1000 UVC days was still observed for a dwell time of 3–6 days [16], in our study, removal at 3–5 days achieved zero CLABSI episodes, still supporting the benefits of UCV early removal. Moreover, this practice is also supported by the median timing of onset of UVC-related CLABSI at 8 (7–12) days of life in the period before bundle, similar to the previously reported onset at a median age of 7–8 days [17].

ECC-related CLABSI were reduced by 64% in the period after bundle (4.4 vs. 12.3/1000 CL days). Because no differences in dwell time and in the proportion of non-elective insertions were observed between the pre- and post-intervention periods, the observed reduction in CLABSI rate is attributable to the efficacy of the bundle. Our results agree with recent studies demonstrating that different bundles for placement and maintenance of ECC were effective in reducing CLABSI in NICU [6,15]. Overall survival analysis considering all ECC-related CLABSI demonstrated no significant differences between the pre- and post-intervention periods. Because the most recent approaches tend to limit the dwell time of ECC to 14 days [19,20], we restricted the analysis to ECC with a dwell time < 14 days and found a significant difference in CLABSI occurrence between the pre- and post-intervention periods. Our results are consistent with efficacy of the bundle in case of dwell time < 14 days, while no beneficial effect is detectable in case of longer dwell time. Interestingly, ECC-related CLABSI occurred after a longer dwell time in the post- vs. pre-intervention period (median 14 vs. 8 days), confirming the bundle’s efficacy during the early phase after ECC placement.

Non-infective complications were reduced by 45% after bundle, but significantly decreased only for ECC, since measures aimed at UVC maintenance were not included in the bundle. The beneficial effect was particularly evident for dislocations, which were reduced by 63%, in agreement with previous studies reporting 85 to 93% decrease with measures that improve stabilization [6,15]. The reduction in non-infective complications is crucial to avoid the need for ECC substitution, and, as a matter of fact, non-elective removal of ECC was reduced by 54% in the post-intervention period.

Our study has some limitations. First, gestational age of enrolled patients ranged from 23 to 42 weeks, not allowing for detection of the bundle impact on subsets of patients with particularly high risk of CLABSI, as those born very preterm. Second, due to staffing issues, CVC were inserted by the dedicated team only in 29% of cases. Third, although real-time ultrasound for tip navigation and location is increasingly recommended to avoid manipulations of CVC [21], it was performed only in 25 and 34% of cases in the period before and after bundle, respectively. Fourth, the number of attempts at CVC insertion was not reported; however, because no differences were found in the expertise of operators inserting CVC between the two periods, no significant difference in performance is expected. Finally, centrally inserted central catheters (CICCs) might be burdened with a reduced risk of CLABSI in comparison to ECCs [19,20], but they were not inserted in the two study periods. The latter two aspects are currently being implemented in our NICU.

## 4. Materials and Methods

### 4.1. Study Design and Patients

This observational retrospective pre–post study was performed at the 3rd-level NICU of Careggi University Hospital, Florence, Italy. We enrolled 2 cohorts of inborn infants of any gestational age admitted to our NICU, who required UVC and/or ECC with dwell time > 48 h. The first cohort included infants born from 1 April 2021 to 31 March 2022; the second cohort included infants born after the implementation of the bundle from 1 September 2022 to 31 August 2023. Infants born in between the two periods were excluded because this intervening time was deemed necessary for bundle implementation in clinical practice. Exclusion criteria were CVC dwell time > 48 h or transfer to other hospitals with an indwelling CVC.

The primary outcome of the study was the number of CLABSI/1000 central line (CL) days. The secondary outcome was the rate of non-infective complications, as dislocation, extravasation/leak, rupture, and occlusion/malfunction of catheters.

### 4.2. Definition and Treatment of CLABSI

CLABSI was defined as a positive blood culture with a CVC in place for at least 48 h, or within the first 48 h after CVC removal, in the absence of any other evident source of infection [22,23]. If blood culture grew Coagulase-negative *Staphylococcus* species, patients were considered as having CLABSI only if C-reactive protein was >10 mg/L (normal value < 5 mg/L) and antibiotics were administered for ≥5 days [22,23]. A second blood culture for confirmation was avoided to limit the risk of iatrogenic anemia [22,23]. Blood samples were obtained from peripheral vein (at least 1 mL) with strict adherence to the aseptic technique [24]. According to local protocol, CLABSI was empirically treated with vancomycin and amikacin followed by targeted antibiotics based on susceptibility of isolates.

At present, no guidelines are available on the criteria for CVC removal during CLABSI in NICU. Therefore, in clinical practice, ECC are not routinely removed in case of CLABSI. For this reason, we decided not to include data on tip culture to support CLABSI diagnosis.

### 4.3. Intervention Bundle

The dedicated team consisted of 3 neonatologists and 3 registered NICU nurses (RN), who performed literature review during a 3-month period. The team was responsible for the education of all involved personnel, defined as any professional role involved in patient care in NICU. The following measures were included in the bundle (Supplementary Table S1).

#### 4.3.1. General Measures

The bundle was presented to all involved personnel during dedicated educational events, and procedures for hand hygiene were also reviewed. Parents are formally educated on hand hygiene on their first entrance into NICU by the attending RN and receive a dedicated information pamphlet.

#### 4.3.2. Antisepsis

Chlorhexidine 2% in isopropyl alcohol in sterile, single-use, hands-off applicators (0.5 mL) was introduced for UVC and ECC insertion [25,26]. In newborns with gestational age < 28 weeks, washing with sterile normal saline is recommended after drying time of chlorhexidine solution [26,27].

#### 4.3.3. Duration of UVCs

Dwell time of UVC should not exceed 5 days [19,20]; longer dwell time up to 7 days could be considered under special circumstances, such as expected poor tolerance to ECC insertion.

#### 4.3.4. Measures to Reduce Intraluminal Contaminations of UVCs and ECCs

Sterile hood with laminar flow is used to prepare IV continuous infusions [19]. Passive disinfection caps containing isopropyl alcohol are used for central line protection [19].

#### 4.3.5. Measures to Reduce Extraluminal Contamination of ECCs

-Cyanoacrylate (CA) glue for securement of ECC [6,15,19,28,29,30]. One or two drops of CA glue are applied at exit site right after insertion of ECC and are re-applied every time that dressing is changed [30]. 

-Adhesive securement device for stabilization of the ECC [6,15,19].

After the previous two steps, a semipermeable transparent membrane is applied, so that the exit site is constantly inspectable for the early identification of local complications or dislocation.

#### 4.3.6. Dedicated Team

For each shift, 3 RNs were included in a dedicated team for the insertion of UVCs and ECCs, together with the medical staff [19,31,32]. All medical staff were included in the team.

All measures not specified in the bundle remained unchanged, particularly policies for antibiotic initiation and antibiotic stopping rule, and criteria for EEC removal.

### 4.4. Statistical Analysis

Based on CLABSI incidence of 16% in the period before the bundle [2,4,5,6], to detect a 60% reduction in CLABSI rate in the period after the bundle [6,12,13,15], with α = 0.05 and power of 0.80, we calculated a sample size of 129 CVC for each cohort.

Data were described as mean and SD, median and IQR, and rate and percentage. Comparisons between groups were performed with Student *t* test for parametric continuous variables, Mann–Whitney U test for continuous nonparametric variables, and Chi-square test for categorical variables. Kaplan–Meier curves for survival free from CLABSI were calculated for the period before and after the bundle.

Because the proportion of newborns < 28 weeks, gender, and delivery mode significantly differed between the two periods, a multivariable logistic regression analysis was performed to assess whether the bundle was a predictive factor for CLABSIs after adjustment for possible confounders.

Data were extracted from electronic charts and were analyzed with SPSS, version 26.0 (IBM, New York, NY, USA).

## 5. Conclusions

In conclusion, we found that the implementation of a bundle for CLABSI prevention in our NICU was effective in reducing the overall CLABSI rate and achieved a significant reduction in both UVC- and ECC-related CLABSI episodes. However, the efficacy for ECC-related CLABSI reduction seems limited to the first 14 days of dwell time. Therefore, adjunctive measures should be considered to further reduce CLABSI rate in NICU, such as improvement of the dedicated team and real-time ultrasound, and proactive vascular planning including different approaches, such as CICC placement, in cases of expected prolonged dwell time.

## Figures and Tables

**Table 1:** Clinical characteristics of patients enrolled before and after implementation of the bundle for CLABSI prevention. Median and (IQR) or number and (%).

	Before Bundle (n = 76)	After Bundle (n = 84)	*p*
Gestational age (wks)	30.0 (26.7–36.3)	31.9 (28–35.6)	0.522
<28 wks	37 (48)	23 (27)	0.005
Birth weight (g)	1190 (850–2467)	1235 (813–2665)	0.435
Females	40 (53)	29 (34)	0.021
Vaginal delivery	38 (50)	24 (29)	0.005
Antenatal steroids	37 (49)	48 (57)	0.284
CRIB II score at admission	6 (1–9)	7 (1–11)	0.059
Respiratory support			
None	12 (15)	16 (19)	0.588
Non-invasive	66 (86)	68 (81)	0.313
Mechanical ventilation	37 (48)	23 (27)	0.284
Surfactant	40 (52)	29 (34)	0.021
Neonatal SOFA score at CLABSI onset	0 (0–5)	0 (0–6)	0.657
hsPDA	24 (32)	15 (17)	0.043
BPD	29 (38)	20 (24)	0.037
NEC	3 (4)	1 (1)	0.370
ROP	2 (3)	0 (0)	0.265
IVH ≥ grade 3	4 (5)	4 (5)	0.827
Hospital stay (d)	52 (14–85)	38 (17–71)	0.061
Death	5 (7)	4 (5)	0.633

BPD, bronchopulmonary dysplasia; hsPDA, hemodynamically significant patent ductus arteriosus; IVH, intraventricular hemorrhage; NEC, necrotizing enterocolitis; ROP, retinopathy of prematurity.

**Table 2:** Characteristics and infective and non-infective complications of UVCs and ECCs before and after bundle. Number and (%).

	Before Bundle (n = 145)	After Bundle (n = 142)	*p*
UVC	67	65	
Duration (d)	8 (6–11)	4 (3–6)	<0.0001
ECC	78	77	
Duration (d)	11 (6–19)	10 (6–17)	0.711
Insertion by dedicated team	0 (0)	41 (29)	<0.0001
US tip navigation/location	36 (25)	48 (34)	0.095
Non-elective removal, total	32 (22)	15 (11)	0.010
UVC	6 (9)	3 (5)	0.492
ECC	26 (33)	12 (15)	0.015
Total CLABSIs, n/1000 CL days	10.7	3.6	0.042
UVC-related, n/1000 UVC days	7.2	0	0.015
ECC-related, n/1000 ECC days	12.3	4.4	0.044
Timing of CLABSI, n. of CL days			
UVC-related	8 (7–12)	N/A	N/A
ECC-related	9 (7–19)	14 (12–19)	0.050
Deaths due to CLABSI	2 (15)	0 (0)	1.000
Non-infective complications	29 (20)	12 (8)	0.033
Dislocation	16 (11)	6 (4)	0.044
Other #	13 (9)	6 (4)	0.153
UVC	10 (7)	10 (7)	1.000
ECC	19 (13)	5 (3)	0.005

N/A, not applicable; US, ultrasound; CL, central line. # Extravasation/leak, Rupture, Occlusion/malfunction.

**Table 3:** Logistic regression analysis for the prediction of CLABSI.

	B	S.E.	Wald	Sig.	Exp (B), 95% C.I.
Bundle	−1.252	0.562	4.961	0.026	0.286 (0.095–0.860)
GA	−0.302	0.106	8.144	0.004	0.739 (0.601–0.910)
Delivery mode	0.403	0.558	0.521	0.470	1.496 (0.501–4.468)
Gender	0.013	0.506	0.001	0.979	1.013 (0.376–2.734)
Constant	6.188	2.813	4.840	0.028	486.639 (-)

GA, gestational age.

## Data Availability

Data that support the findings of this study are available from the corresponding author upon reasonable request.

## Supplementary Materials

The following supporting information can be downloaded at: https://www.mdpi.com/article/10.3390/antibiotics14121208/s1, Table S1. Summary of differences between period before and after bundle. Table S2. Personnel performing CVC insertion in the period before and after bundle.

## Abbreviations

The following abbreviations are used in this manuscript:

CLABSI	Central Line-Associated Bloodstream Infections
CVC	Central Venous Catheter
ECC	Epicutaneo-Caval Catheter
NICU	Neonatal Intensive Care Unit
UVC	Umbilical Venous Catheter
