# Supplementary material for: A Quality Improvement Bundle to Reduce Central Line-Associated Bloodstream Infections in Neonatal Intensive Care Unit: An Observational Study
Source: Antibiotics (Basel). 2025 Dec 1;14(12):1208. doi: 10.3390/antibiotics14121208 (PMC12729402; doi:10.3390/antibiotics14121208)
Supplement: Supplementary file 1 [file antibiotics-14-01208-s001.zip › antibiotics-3987680-supplementary.pdf]

**Supplementary Table S1.** Summary of differences between period before and after bundle.

| Before bundle | After bundle | Reasons for change |
|---|---|---|
| **General measures** | | |
| Hand hygiene and education:<br>no specific interventions | Presentation of the bundle and refresh on hand hygiene to all personnel<br>Visitors' education/informative material | Education on CLABSI and reinforcement of hand hygiene effective to reduce CLABSI rate [12-14,,19] |
| **Antisepsis** | | |
| UVC: Iodopovidone | UVC and ECC: Chlorhexidine 2% in IPA - sterile single use 0.5 mL applicators<br>*(Chloraprep™, BD, USA)* | Chlorhexidine has higher and faster bactericidal efficacy [19-25]<br>Sterile single dose applicators avoid a. undesired pooling of solution, responsible for skin lesions b. the risk of bacterial contamination of the antiseptic solution [19,25] |
| ECC: Chlorhexidine 2% in IPA<br>not single use applicators | | |
| **Duration of UVC** | | |
| Up to 14 days | Up to 5 days | Dwell time of 3 to 7 days might be a standard of practice to minimize CLABSI [19,20] |
| **Measures to reduce extraluminal contamination of ECCs** | | |
| Steri-strip on exit site | CA glue, 1-2 drops, on exit site<br>To be reapplied when medication is changed<br>*(Glubran Tiss 2, 0.25 mL, GEM, Italy)* | CA glue was included in effective bundles for CLABSI prevention [6,15,19]<br>CA glue reduces extraluminal contamination by a. sealing the exit-site and reducing in and out micromovements, which prevents bacterial translocation; b. enhancing hemostasis, which disfavors bacterial growth; c. possible antibacterial activity [28,29]<br>No detrimental effects on polyurethan CVC, despite long-term contact [30] |
| Steri-strip along the ECC | Adhesive securement device<br>*(Griplock, Vygon, France)* | Further stabilization of ECC, reducing in and out micromovements [6,15,19] |
| **Measures to reduce intraluminal contamination of UVCs and ECCs** | | |
| Preparation of all IV infusions with sterile technique, no hood | Preparation of continuous IV infusions under sterile hood with laminar flow | Sterile hood might contribute to maintain sterility of solutions [19] |
| "Scrub the hub" for 15 sec with chlorhexidine 2% in IPA, when the line is accessed | Passive disinfection caps, containing IPA<br>To be applied to NFC, to male Luer connectors, to CVC hub<br>*(Dual Cap ® Solo™, Merit Medical, US Curos™ Stopper, 3M™, US)* | Caps induce effective bacterial killing in 1 minute [19]<br>Caps are considered more effective than the "scrub the hub" because they avoid possible variability of the procedure [19] |
| **Dedicated team** | | |
| No dedicated personnel | All medical staff and 15 RNs included in the vascular access team | Dedicated team effective to reduce complications of CVC [6,13,19,31,32] |

CA, cyanoacrilate; CVC, central venous catheter; ECC, epicutaneo-caval catheter; IPA, isopropyl alcohol; NFC, needle free connector; UVC, umbilical venous catheter

**Supplementary Table S2.** Personnel performing CVC insertion in the period before and after bundle.

| | Fellow | Neonatologist | *p* |
|---|---|---|---|
| UVC | | | |
| Before (n=67) | 22 | 45 | 0.572 |
| *Dedicated team* | -- | *0* | |
| After (n=65) | 18 | 47 | *<0.0001 for dedicated team* |
| *Dedicated team* | -- | *18* | |
| ECC | | | |
| Before (n=78) | 6 | 72 | 0.745 |
| *Dedicated team* | -- | *0* | |
| After (n=77) | 4 | 73 | *<0.0001 for dedicated team* |
| *Dedicated team* | -- | *23* | |
